# Mechanisms of resistance to antibody-drug conjugates in bladder cancer

**DOI:** 10.3389/fphar.2026.1882868

**Published:** 2026-08-17

**Authors:** Kai Liu, Feixiang Xu, Chenyang Wu, Miao Wang

**Affiliations:** Department of Urology, Union Hospital, Tongji Medical College, Huazhong University of Science and Technology, Wuhan, China

**Keywords:** antibody-drug conjugates, bladder cancer, combination therapy, drug resistance, resistance mechanisms

## Abstract

Antibody-drug conjugates (ADCs) have made important advances in the treatment of bladder cancer (BC) in recent years and have demonstrated significant clinical benefit in locally advanced or metastatic urothelial carcinoma (UC). However, the emergence of resistance remains a key factor limiting the durability of their therapeutic efficacy. Current evidence indicates that resistance to ADCs in BC is a complex process driven by multiple tumor-intrinsic and tumor-extrinsic factors. This review systematically summarizes the major mechanisms underlying ADC resistance in BC and outlines potential strategies to overcome it, including the development of next-generation ADCs, combination therapies, and predictive biomarkers. A deeper understanding of BC-specific mechanisms of ADC resistance will help promote the development of more precise and individualized ADC-based treatment strategies.

## 1 Introduction

According to the most recent data from the GLOBOCAN online database, bladder cancer (BC) ranks as the ninth most commonly diagnosed malignancy worldwide and the sixth most common malignancy among men (Bray et al., 2024). Approximately 75% of BC cases are classified as non-muscle-invasive bladder cancer (NMIBC), whereas the remaining 25% are muscle-invasive bladder cancer (MIBC). Owing to advances in therapeutic strategies, most patients with NMIBC currently achieve favorable outcomes, with a 5-year overall survival (OS) rate exceeding 90%. However, the 5-year postoperative recurrence rate of NMIBC remains as high as 31%–78%, and high-grade NMIBC is prone to invasion and progression to MIBC (Lobo et al., 2022; Lopez-Beltran et al., 2024). Currently, BC treatment is determined largely by disease stage. NMIBC is generally managed with transurethral resection of bladder tumor (TURBT), followed by risk-adapted intravesical therapy such as chemotherapy or *bacillus* Calmette–Guérin (BCG). By contrast, MIBC usually requires more intensive multimodal treatment, including radical cystectomy with pelvic lymph node dissection, cisplatin-based neoadjuvant chemotherapy for eligible patients, perioperative systemic therapy, or bladder-preserving trimodality therapy in selected cases (Witjes et al., 2020; Nadal et al., 2024).

ADCs have rapidly become an important therapeutic modality in oncology. By combining antibody-mediated target specificity with highly potent cytotoxic payloads, ADCs can promote selective tumor-cell killing while potentially limiting systemic toxicity (Wang Y. et al., 2025). In BC, the clinical impact of ADCs has been most evident in UC, particularly in locally advanced or metastatic disease, and their use is now extending into perioperative MIBC settings. Accordingly, the subsequent discussion mainly focuses on MIBC and advanced or metastatic UC, whereas NMIBC is discussed only when prior intravesical treatment or local urothelial exposure is relevant to ADC resistance. Enfortumab vedotin (EV) was the first ADC approved for UC. Its initial FDA approval in 2019 was for patients with locally advanced or metastatic UC who had previously received a programmed cell death protein 1 (PD-1) or programmed death-ligand 1 (PD-L1) inhibitor and platinum-based chemotherapy in the neoadjuvant/adjuvant, locally advanced, or metastatic setting (Rosenberg et al., 2019; Chang et al., 2021). The clinical role of EV has since expanded substantially. EV plus pembrolizumab first received accelerated approval for cisplatin-ineligible patients with locally advanced or metastatic UC based on EV-103 (Maguire et al., 2024) and later received traditional approval as first-line therapy for locally advanced or metastatic UC based on EV-302 (Brave et al., 2024; Powles et al., 2024). More recently, perioperative EV plus pembrolizumab has shown significant clinical benefit in MIBC, further extending ADC-based therapy from advanced disease into earlier treatment settings (Vulsteke et al., 2026). These developments highlight the expanding clinical importance of ADCs in BC, but durable disease control remains limited (Singh et al., 2023; Zhang and Li, 2025). The molecular and cellular mechanisms underlying ADC resistance in BC remain incompletely characterized. Defining these mechanisms is essential for refining treatment strategies and maximizing clinical benefit. Recent and ongoing clinical trials of ADCs in UC are summarized in Table 1.

**TABLE 1:** Recent and ongoing clinical trials of ADCs in urothelial carcinoma.

Target	ADC	Condition	Combination	Trial phase	Estimated completion date	NCT number
Nectin-4	EV	La/mUC	None	Phase II	2023	NCT03219333
		La/mUC	None	Phase III	2025	NCT03474107
		La/mUC	Cabozantinib	Phase I	2026	NCT04878029
		MIBC	Pembrolizumab	Phase II	2027	NCT05239624
		La/mUC	Pembrolizumab	Phase II	2029	NCT07221942
HER2	DV	La/mUC	None	Phase II	2023	NCT03809013
		MIBC	Toripalimab	Phase I/II	2027	NCT06341400
		La/mUC	Toripalimab	Phase III	2028	NCT05302284
		La/mUC	−/+Pembrolizumab	Phase II	2029	NCT04879329
		La/mUC	Pembrolizumab	Phase III	2029	NCT05911295
Trop-2	SG	UC	None	Phase II	2025	NCT05226117
		MIBC	Nivolumab	Phase II	2026	NCT06682728
		UC	EV	Phase II	2028	NCT04724018
			−/+Pembrolizumab			
		MIBC	Zimberelimab Domvanalimab	Phase II	2030	NCT06133517

Abbreviations: ADC, antibody-drug conjugate; Nectin-4, nectin cell adhesion molecule 4; HER2, human epidermal growth factor receptor 2; Trop-2, trophoblast cell surface antigen 2; EV, enfortumab vedotin; DV, disitamab vedotin; SG, sacituzumab govitecan; UC, urothelial carcinoma; la/mUC, locally advanced or metastatic urothelial carcinoma; MIBC, muscle-invasive bladder cancer; NCT, national clinical trial identifier.

Although understanding the mechanisms of action of ADCs is important, detailed reviews on their basic principles are already available (Schrama et al., 2006; Sievers and Senter, 2013; Fu et al., 2022; Tsuchikama et al., 2024; Zhou K. et al., 2025). Therefore, this review does not systematically recapitulate general ADC biology but focuses on resistance to ADCs in BC.

## 2 Mechanisms of resistance to ADC therapy

Despite their molecular specificity and targeted drug delivery, ADCs are not exempt from the development of therapeutic resistance under prolonged exposure or selective pressure. Clinically, as with other systemic therapies, resistance to ADCs can be classified into primary resistance and acquired resistance. Primary resistance refers to absent or minimal response after ADC initiation, whereas acquired resistance refers to progression after an initial response or disease control. Notably, research on acquired resistance to ADC therapy remains at an early stage. Moreover, ADC resistance is multifactorial, involving multiple processes and biological levels, and mechanisms underlying primary and acquired resistance may partially overlap (Shefet-Carasso and Benhar, 2015; Li et al., 2025).

**FIGURE 1:**
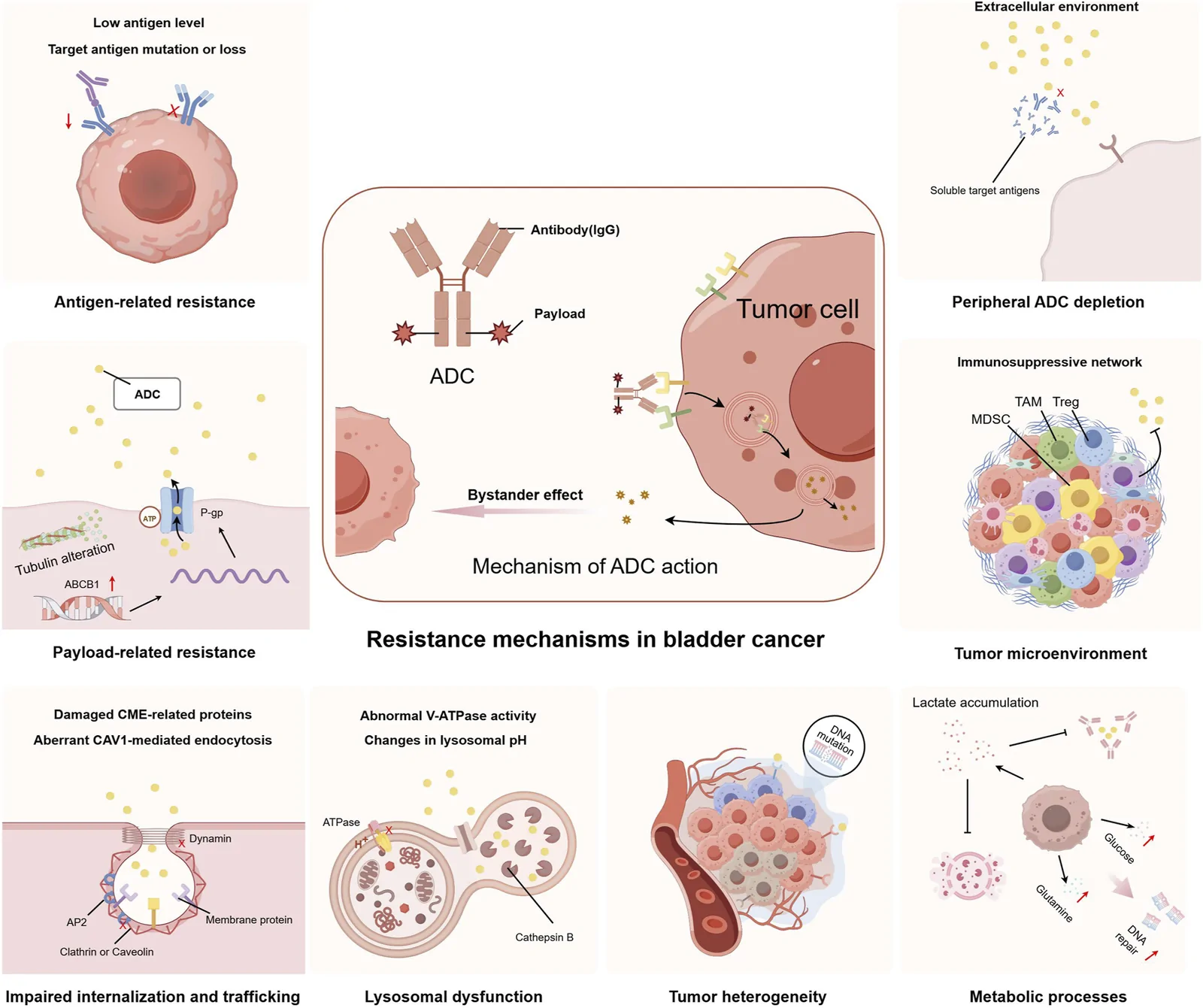
Schematic overview of the major mechanisms contributing to resistance to ADCs in BC. The central panel illustrates the mechanism of ADC action, including target binding, internalization, intracellular payload release, tumor cell killing, and the bystander effect. The surrounding panels summarize the principal resistance pathways discussed in this review, including antigen-related resistance, impaired internalization and trafficking, lysosomal dysfunction, payload-related resistance, tumor heterogeneity, peripheral ADC depletion, the tumor microenvironment, and metabolic processes. These mechanisms may independently or cooperatively limit the efficacy of ADC therapy in BC. Abbreviations: ADC, antibody–drug conjugate; BC, bladder cancer; TME, tumor microenvironment; TAM, tumor-associated macrophage; Treg, regulatory T cell; MDSC, myeloid-derived suppressor cell; CME, clathrin-mediated endocytosis; CAV1, caveolin-1; P-gp, P-glycoprotein.

Accordingly, we focus on the established and putative mechanisms of resistance to ADC therapy in BC. Based on the mechanisms of action of ADCs, we broadly categorize resistance into two classes: tumor-intrinsic and tumor-extrinsic mechanisms (Figure 1). Tumor-intrinsic mechanisms include antigen-related resistance, impaired internalization and trafficking, lysosomal dysfunction, payload-related resistance, and tumor heterogeneity. Tumor-extrinsic mechanisms include peripheral ADC depletion and the tumor microenvironment (TME). Metabolic processes are discussed as cross-cutting modulators because they may affect both tumor-cell payload sensitivity and microenvironment-mediated ADC delivery or immune synergy. Importantly, although many resistance mechanisms proposed for ADCs have been extrapolated from other solid tumors, the unique biological context of BC—including its molecular heterogeneity, prior intravesical treatment history, urinary exposure, and distinct microenvironmental and metabolic landscape (Bevers et al., 2004; Biswas et al., 2023; Zhang et al., 2024; Trần et al., 2025)—may shape resistance in a disease-specific manner. For this reason, the mechanisms discussed below are interpreted according to the strength and source of evidence. Target antigen heterogeneity, particularly Nectin-4-related resistance to EV, is supported by BC-specific clinical or translational evidence, whereas P-glycoprotein (P-gp)-mediated payload efflux, impaired intracellular trafficking, lysosomal dysfunction, and metabolic regulation of ADC sensitivity are mainly supported by non-BC or pan-cancer ADC studies. Unless direct BC evidence is available, these mechanisms are therefore discussed as biologically plausible and BC-relevant hypotheses rather than established BC-specific resistance pathways.

### 2.1 Tumor-intrinsic mechanisms

#### 2.1.1 Antigen-related resistance

##### 2.1.1.1 Nectin-4

Nectin-4 is a transmembrane cell-adhesion molecule of the nectin family that is frequently expressed on BC cells. Beyond serving as the target of EV, Nectin-4 has been implicated in tumor cell adhesion, proliferation, invasion, and immune-related processes, and its expression has been associated with aggressive tumor behavior and poor prognosis in several malignancies (Bouleftour et al., 2023). In BC, heterogeneous or reduced Nectin-4 expression is particularly relevant because it may limit EV binding, internalization, and subsequent payload delivery. A key distinction should be made between tumors with low or absent Nectin-4 expression before treatment and tumors that initially express Nectin-4 but later lose or reduce its expression. Tumors lacking sufficient Nectin-4 at treatment initiation would be expected to have limited sensitivity to EV because target engagement is inadequate. This situation is more consistent with primary resistance or intrinsic non-response. By contrast, reduced Nectin-4 expression after EV exposure or during disease progression may contribute to acquired resistance in tumors that were initially target-positive and responsive. Preclinical evidence supports the dependence of EV sensitivity on Nectin-4 expression. Chu et al. showed that lack of Nectin-4 expression was associated with resistance to EV in BC models (Chu et al., 2021). In a retrospective clinical study, Klümper et al. reported that membranous Nectin-4 expression frequently decreased during metastatic progression of UC; 59.1% of patients showed a marked decrease in Nectin-4 expression, and 39.4% of metastatic tumors lacked Nectin-4 expression. Low or absent membranous Nectin-4 expression was also associated with shorter progression-free survival (PFS) during EV treatment (Klümper et al., 2023). In another study, prolonged exposure to EV led BC cells to downregulate Nectin-4 and acquire resistance to EV (Chou et al., 2022). In addition, Wu and colleagues showed that α2,6-sialylation mediates Nectin-4 downregulation and that targeting sialylation enhances EV efficacy in BC (Wu et al., 2026). Together, these data suggest that reduced Nectin-4 expression may contribute to resistance to Nectin-4-targeted ADC therapy in BC.

It should also be clarified that EV is not routinely administered only to patients selected by Nectin-4 immunohistochemistry. Clinical approvals and approval summaries have defined the eligible population primarily by disease setting and prior treatment history rather than by mandatory Nectin-4 companion diagnostic testing (Chang et al., 2021; Brave et al., 2024). Therefore, Nectin-4 expression may help explain differential sensitivity or resistance, but it is not currently used as a required selection criterion for EV treatment in routine clinical practice.

##### 2.1.1.2 HER2

HER2 is a growth-promoting receptor tyrosine kinase that is expressed at low levels or is absent under physiological conditions but is frequently overexpressed in several solid tumors, where it contributes to cellular proliferation and tumorigenesis. As both a prognostic factor and a therapeutic target, HER2 has been extensively studied in breast and gastric cancers, and anti-HER2 therapy has become a cornerstone of treatment for these malignancies (Joshi and Badgwell, 2021; Marra et al., 2024). In BC, HER2 protein overexpression or gene amplification has also been reported and may be relatively frequent in selected disease settings (Raggi et al., 2026). Available preclinical and clinical evidence has positioned HER2-directed ADCs as an important therapeutic strategy in HER2-expressing UC (Patelli et al., 2022). Disitamab vedotin (DV), a HER2-directed ADC, has been approved in China for patients with HER2-overexpressing locally advanced or metastatic UC who have previously received platinum-based chemotherapy (Deeks, 2021). More recently, a phase III clinical study showed that DV plus toripalimab improved clinical outcomes compared with chemotherapy in previously untreated patients with HER2-expressing locally advanced or metastatic UC (Sheng et al., 2025). These data support HER2 as a clinically relevant ADC target in BC.

However, HER2-related resistance should be interpreted cautiously. HER2 expression may be heterogeneous across primary and metastatic lesions or among tumor subclones, potentially limiting ADC binding and payload delivery in HER2-low or HER2-negative regions. In addition, altered cell-surface HER2 and HER2-related signaling have been implicated in ADC resistance in other tumor types (Chen et al., 2023; Passaro et al., 2023). Simon et al. reported that HER2 amplification is frequently associated with genomic alterations in TOP2A in BC (Simon et al., 2003), and TOP2A overexpression has been linked to BC cell survival, proliferation, and invasion (Zeng et al., 2019). These findings suggest that HER2/TOP2A-related alterations may be relevant to the biology of HER2-positive BC. However, direct evidence linking these alterations to response or resistance to HER2-targeted ADCs in BC remains limited, and their predictive value requires further validation in ADC-treated cohorts.

##### 2.1.1.3 Trop-2

Trop-2 is a transmembrane glycoprotein encoded by TACSTD2 and is widely expressed in several epithelial malignancies, including BC. Sacituzumab govitecan (SG) is a Trop-2-directed ADC composed of an anti-Trop-2 monoclonal antibody linked to SN-38, the active metabolite of irinotecan (Tagawa et al., 2021). Trop-2 is therefore a therapeutically relevant ADC target in UC. In BC, heterogeneous or dynamic Trop-2 expression may influence target engagement and ADC sensitivity, although the relationship between Trop-2 expression and clinical response remains complex (Qiu et al., 2023; Loriot et al., 2024a). In EV-resistant BC models, Chou et al. found that prolonged EV exposure led to Nectin-4 downregulation, while Trop-2 expression was retained and TROP2 mRNA levels exceeded NECTIN4 mRNA levels. These cells remained sensitive to Trop-2-targeted ADCs (Chou et al., 2022). This finding raises the possibility that, in selected EV-resistant BC models, loss of Nectin-4 may coexist with retained or increased Trop-2 expression. However, whether this represents a clinically relevant adaptive target-switching mechanism in patients with BC remains unclear. Therefore, Trop-2–targeted ADCs may provide a rational post-EV therapeutic strategy in selected contexts, but this hypothesis requires further clinical validation.

Clinically, SG has shown activity in previously treated advanced UC. A pivotal multicenter phase III clinical trial showed that SG achieved a higher objective response rate (ORR) in previously treated advanced UC, although this did not translate into a survival advantage because of substantial early toxicity, particularly infectious complications (Powles et al., 2025b). These findings indicate that Trop-2-directed ADC therapy remains biologically and clinically relevant in BC, but its optimal use requires careful patient selection, toxicity monitoring, and further clarification of Trop-2-related resistance mechanisms.

#### 2.1.2 Impaired internalization and trafficking

Following binding to surface antigens on tumor cells, ADCs exert their effects after entering tumor cells via endocytosis. Endocytosis can occur through multiple pathways, including clathrin-mediated endocytosis (CME), caveolae-mediated endocytosis (CDE), and clathrin and caveolae-independent endocytosis. CME is dependent on clathrin, which facilitates inward invagination and budding of the plasma membrane, ultimately forming vesicles that transport ADC-antigen complexes into the cell (Banushi et al., 2023; Sung et al., 2025). By analogy with other ADC models, alterations in CME-associated proteins, such as clathrin, adaptor protein 2 (AP2), and dynamin, could theoretically reduce ADC internalization and intracellular delivery. In BC, however, direct evidence linking these trafficking abnormalities to clinical ADC resistance remains limited. Caveolin-1 (CAV1), a key protein involved in CDE, is closely associated with cargo selection and the rate of internalization. Current evidence suggests that CME is a major route utilized by many ADCs, through which cargo is efficiently trafficked to lysosomes for degradation (Kalim, 2017). However, in resistant cancer cells, ADCs have been found to colocalize with CAV1, while their colocalization with lysosomes is markedly reduced (Smith et al., 2006). One study reported that CAV1-mediated internalization of trastuzumab emtansine (T-DM1) is associated with reduced ADC responsiveness (Sung et al., 2018). CAV1 is a key protein involved in CDE and can affect cargo selection and internalization kinetics. Therefore, altered endocytic routing may reduce ADC efficacy by diverting ADC-antigen complexes away from productive lysosomal processing.

Wu et al. reported that sialylation influences the therapeutic efficacy of EV in BC, and that targeting sialylation enhances EV internalization in BC cells, thereby improving treatment efficacy (Wu et al., 2026). This provides a new perspective on ADC resistance arising from impaired endocytosis. Taken together, these findings suggest that BC cells with defective internalization and trafficking pathways may be more resistant to ADC therapy; however, further studies are needed to clarify how abnormalities in these pathways contribute to resistance to ADC treatment.

#### 2.1.3 Lysosomal dysfunction

ADCs ultimately need to be trafficked to lysosomes for degradation in order to release their cytotoxic payloads. If lysosomal function is impaired—for example, because of insufficient enzymatic activity or abnormal pH—payload release is likely to be compromised, thereby leading to resistance. The acidic pH of lysosomes is maintained by vacuolar H + -ATPase (V-ATPase), which pumps protons from the cytoplasm into the lysosomal lumen. Moreover, lysosomes harbor a variety of hydrolases with specificity for different substrates, and these enzymes are activated by the highly acidic intralysosomal environment (Mindell, 2012). It has been reported that aberrant lysosomal V-ATPase activity in T-DM1-resistant tumors reduces the generation of T-DM1 catabolites, ultimately resulting in resistance to T-DM1. Treatment of T-DM1-sensitive tumor cells with the selective V-ATPase inhibitor bafilomycin A1 can induce T-DM1 resistance, further indicating that abnormal V-ATPase activity impairs T-DM1 metabolism and thereby promotes resistance. Notably, HER2-targeted ADCs containing protease-cleavable linkers appear to be less affected by this mechanism (Wang et al., 2017). We speculate that this may be because protease-cleavable linkers can be cleaved by lysosomal cathepsin B, thereby releasing the membrane-permeable payload. However, some studies have shown that even when cathepsin B levels are reduced, the efficacy of the corresponding ADCs is not significantly affected (Caculitan et al., 2017). This suggests that multiple pathways may exist for the generation of active catabolites from these linkers, thereby avoiding ADC resistance caused by abnormalities in cathepsin B. In another study, Luci et al. likewise found that an elevated lysosomal pH and impaired lysosomal proteolytic activity contributed to ADC resistance (Ríos-Luci et al., 2017). Notably, to improve the efficacy of ADCs in tumor treatment, DeVay and colleagues designed a bispecific ADC in which one arm targeting HER2 directs the ADC to the tumor, whereas the other arm targets amyloid precursor-like protein 2 (APLP2) with the aim of facilitating lysosomal trafficking for payload release. The results showed that APLP2-targeted lysosome-directing ADCs were more effective than Trop-2–targeted ADCs in both *in vitro* and *in vivo* settings (DeVay et al., 2017). To some extent, this strategy may alleviate resistance to ADC therapy arising from defective lysosomal trafficking; however, it is still insufficient to address the fundamental problem of ADC resistance and requires further clinical validation. In addition, several studies have explored the enhancement of chemotherapeutic efficacy through lysosome-related pathways, thereby providing potentially useful therapeutic strategies for BC. Nevertheless, whether these approaches can be applied to overcome ADC resistance remains uncertain (Jin et al., 2017; Hou et al., 2022; Kong et al., 2025). In summary, lysosomal dysfunction is a well-supported mechanism of ADC resistance in several models, particularly for ADCs that require lysosomal degradation for payload release. For EV, DV, and other ADCs used in BC, this mechanism is biologically plausible, but direct BC-specific evidence remains limited. Further studies are needed to determine whether impaired lysosomal hydrolytic activity or defective lysosomal trafficking contributes to clinically relevant ADC resistance in BC.

#### 2.1.4 Payload-related resistance

The payload is a critical component of the antitumor activity of ADCs and plays a central role in tumor cell killing. At present, the payloads used clinically in ADC therapy for BC are predominantly MMAE-based. MMAE is a microtubule inhibitor that binds to intracellular tubulin, prevents tubulin polymerization, and disrupts the stability of the microtubule network. Owing to its potent cytotoxic activity, MMAE is widely used as a payload in ADC-based cancer therapy (Tiwatane et al., 2025). Sauveur et al. identified complex modifications of the tubulin complex in tumor cells resistant to trastuzumab emtansine (T-DM1). Qualitative or quantitative alterations in tubulin may affect drug binding, thereby potentially diminishing the efficacy of microtubule inhibitors and contributing to ADC resistance (Dumontet and Jordan, 2010; Sauveur et al., 2020). These mechanisms are mainly supported by non-BC models, but they are relevant to MMAE-based ADCs used in BC.

Drug efflux is another payload-related mechanism. ABCB1 is the gene encoding P-gp, an ATP-binding cassette transporter that can export several cytotoxic agents from tumor cells (Tian et al., 2023). In ADC therapy, P-gp-mediated efflux is expected to be most relevant after intracellular payload release, because intact ADCs are not typical P-gp substrates. Thus, MMAE must generally be released from the ADC complex, or generated as a diffusible cytotoxic catabolite, before it can be recognized and effluxed by P-gp. In non-Hodgkin lymphoma, Yu et al. found that ABCB1 upregulation was a major driver of resistance to MMAE-based conjugates (Yu et al., 2015). In a preclinical murine cancer model, Cabaud et al. showed that ABCB1 expression was upregulated in resistant tumors and that pharmacological P-gp inhibition restored sensitivity to Nectin-4-targeted ADCs *in vitro* and *in vivo* (Cabaud et al., 2022). Because MMAE-based ADCs are used in BC, ABCB1/P-gp-mediated efflux is biologically plausible in this setting, but its clinical relevance in ADC-treated BC remains insufficiently validated.

Additional payload-related factors may also influence ADC response. Recent studies showed that homologous recombination-deficient BC cells exhibit increased sensitivity to MMAE, possibly through enhanced apoptotic signaling in the context of DNA damage (Shanmugam et al., 2025). In a multicenter retrospective study of patients treated with EV and other agents, low tumor mutational burden (TMB) was associated with shorter OS (Jindal et al., 2023). One possible explanation is that low TMB may limit immunogenic cell death and reduce immune amplification after EV-mediated tumor-cell killing. Overall, payload-related resistance in BC may involve altered payload target sensitivity, reduced intracellular payload accumulation, drug efflux after payload release, and limited immune amplification, but several of these mechanisms still require BC-specific validation.

#### 2.1.5 Tumor heterogeneity

Tumor heterogeneity refers to the diversity and variation among tumor cells within the same tumor with respect to genotype, phenotype, and behavioral characteristics, such as proliferative capacity, metastatic potential, and response to therapy (Wu and Sheng, 2024; Dagogo-Jack and Shaw, 2018). In particular, for anticancer agents that depend on antigen expression for target recognition and activity, such as ADCs, heterogeneity-driven variation in antigen expression is likely to contribute to therapeutic resistance (Chen B. et al., 2025). For example, Nectin-4 expression is heterogeneous across the molecular subtypes of BC and is significantly enriched in the luminal subtype. This variability has been associated with differential sensitivity to EV, whereas downregulation of Nectin-4 on the tumor cell surface may contribute to resistance (Chu et al., 2021; Hoffman-Censits et al., 2021). These findings indicate that the expression of relevant antigenic molecules differs across tumor subtypes, and that certain molecular subtypes may be more prone to resistance. In addition, in a study of HER2-targeted ADC therapy, patients with HER2 amplification appeared to derive greater benefit from DV treatment than those without amplification (61.5% vs. 44.8%, P = 0.059). Notably, however, there was no significant difference in ORR between patients with tumor heterogeneity (55.5%) and those with homogeneous tumors (Lei et al., 2023). This may be attributable to the relatively small number of patients included in the study, or it may reflect tumor adaptation to the host immune response. Regardless, the potential benefits and challenges of HER2 expression heterogeneity in HER2-targeted ADC therapy for BC warrant further investigation. Tumor heterogeneity is likely to be a central determinant of ADC resistance in BC, although the extent to which it drives subtype-specific therapeutic failure remains incompletely defined.

### 2.2 Tumor-extrinsic mechanisms

#### 2.2.1 Peripheral ADC depletion

In ADC therapy for tumors, soluble target antigens present in the periphery or within the tumor microenvironment may act as “decoys” by binding to the drug, thereby reducing its effective delivery to tumor tissue. This phenomenon has emerged as a potential mechanism affecting ADC efficacy in some therapeutic settings (van der Velden et al., 2004). This mechanism could also be relevant to BC, although its quantitative contribution to ADC exposure, tumor delivery, and clinical resistance has not been clearly established. Taking EV, which targets Nectin-4, as an example, soluble Nectin-4 can be detected in the blood and urine of patients with BC (Heath and Rosenberg, 2021; Miyake et al., 2023). These free antigens could theoretically bind ADCs and reduce the fraction of drug available for tumor targeting, but direct pharmacokinetic and clinical evidence supporting this mechanism in BC remains limited. Similarly, other BC-related targets, such as Trop-2 and HER2, may also be present in the circulation and tumor microenvironment in soluble or vesicle-associated forms through mechanisms such as exosomal secretion or proteolytic shedding. These forms may subsequently neutralize ADCs and hinder their effective binding to target antigens on the tumor cell surface (Ciravolo et al., 2012; Tomiyama et al., 2022). At present, it remains unclear whether BC cells actively mediate adaptive resistance to ADCs by regulating exosome release or antigen shedding. This mechanism warrants further investigation to clarify how tumor-associated antigen depletion affects ADC biodistribution, target engagement, and therapeutic efficacy.

#### 2.2.2 TME

In BC, the TME may influence ADC efficacy through several pharmacologic and immunologic steps (Lee et al., 2022), including tumor delivery, tissue penetration, payload-mediated killing, bystander effects, and synergy with immune checkpoint inhibition (Joyce and Fearon, 2015; Alfano et al., 2016). However, the extent to which each component directly drives ADC resistance in patients with BC remains incompletely defined.

At the tissue level, stromal remodeling, extracellular matrix deposition, and abnormal vascular architecture can restrict ADC delivery and intratumoral penetration, thereby reducing the amount of antibody-bound payload that reaches antigen-positive tumor cells. At the cellular level, heterogeneous drug distribution may leave poorly perfused or stromal-rich tumor regions insufficiently exposed, creating spatial niches for residual disease. In MIBC, marked stromal fibrosis and dense extracellular matrix deposition may therefore represent an important barrier to the homogeneous distribution of macromolecular drugs such as ADCs (Tannock et al., 2002; Child et al., 2025; Huang P. et al., 2025). This spatial drug-distribution problem is also relevant to bystander killing. For ADCs carrying membrane-permeable payloads, released cytotoxic agents can diffuse from antigen-positive cells to adjacent antigen-low or antigen-negative cells. However, the extent of this bystander effect depends on payload permeability, local payload concentration, tissue penetration, and the spatial arrangement of sensitive and resistant tumor cells (Khera et al., 2022). Modeling studies suggest that heterogeneous receptor expression and heterogeneous ADC distribution can promote the selection of resistant cells even when bystander payloads are present (Menezes et al., 2022). These findings are not BC-specific, but they provide a useful mechanistic explanation for why stromal-rich, poorly perfused, or antigen-heterogeneous BC lesions may show incomplete responses to ADC therapy.

The immune component of the BC TME may further determine whether ADC-induced tumor-cell death is converted into durable antitumor immunity. BC tissues can contain multiple immunosuppressive cell populations, including tumor-associated macrophages (TAMs), regulatory T cells (Tregs), and myeloid-derived suppressor cells (MDSCs) (Schneider et al., 2019). Within the TME, these cells may not directly prevent ADC binding to target antigens, but they can reduce the downstream immune consequences of ADC activity by suppressing cytotoxic T-cell function, impairing antigen presentation, and limiting the amplification of immunogenic cell death (Chau et al., 2019; Jing et al., 2024; Chen Z. et al., 2025). This issue is particularly relevant to ADC-immunotherapy combinations. In urothelial cancer, stromal and EMT-related gene expression has been associated with resistance to PD-1 blockade (Wang et al., 2018), and a T-cell-to-stroma enrichment score has been shown to predict response to immune checkpoint inhibitors (ICIs) in urothelial cancer (Rijnders et al., 2024). These findings support the concept that the balance between immune infiltration and stromal exclusion can influence the efficacy of immune-based therapy in BC.

ADCs may partially counteract this suppressive state by inducing immunogenic cell death and promoting antigen release, dendritic-cell activation, and T-cell recruitment (Tan et al., 2023; Gedik et al., 2024; Shi et al., 2025). However, if the BC TME is dominated by stromal exclusion, hypoxia, or suppressive myeloid populations, this immune conversion may be attenuated. Hypoxia is especially relevant because it has been associated with both immune infiltration and immune-suppressive signaling in MIBC (Smith et al., 2023). Therefore, TME-mediated ADC resistance in BC may involve a linked sequence: restricted ADC delivery, heterogeneous internalization and payload release, incomplete bystander killing, and reduced conversion of ADC-induced cell death into effective antitumor immunity.

### 2.3 Metabolic processes as cross-cutting modulators

Metabolic processes are discussed here as cross-cutting modulators of ADC efficacy rather than as an entirely separate resistance category. In BC, the TME is characterized by enhanced glycolysis, lactate accumulation, hypoxia, extracellular acidosis, and intense nutrient competition (Patel et al., 2019; Schmidt et al., 2021; Peng et al., 2023). These features may influence ADC therapy through several interconnected mechanisms. Hypoxia and abnormal tumor perfusion may reduce ADC delivery to poorly vascularized tumor regions, thereby reinforcing spatial heterogeneity in drug exposure. Lactate accumulation and extracellular acidosis may affect antibody-antigen binding or ADC stability at the tumor-cell surface, although this possibility remains incompletely validated for BC-specific ADC targets such as Nectin-4, Trop-2, and HER2 (Klaus and Deshmukh, 2021; Xing et al., 2025). Metabolic stress may also alter endolysosomal homeostasis and linker processing after ADC internalization, potentially affecting payload release (Li et al., 2025). These mechanisms provide a biochemical link between the metabolic TME and ADC pharmacology, but direct evidence in ADC-treated BC cohorts remains limited.

Metabolic reprogramming may affect the sensitivity of tumor cells to released payloads. Enhanced glycolysis and glutamine metabolism can support redox balance, nucleotide synthesis, and stress adaptation, which may increase tolerance to oxidative stress, DNA damage, or microtubule disruption induced by ADC payloads (Ritterson Lew et al., 2015; Shigeta et al., 2023). This effect may influence antigen-positive cells that directly internalize ADCs, as well as antigen-low neighboring cells that are killed by released payloads through the bystander effect. If metabolically adapted tumor regions are less susceptible to cytotoxic injury, bystander killing may be weakened even when payload diffusion occurs (Guo et al., 2024). Therefore, metabolic heterogeneity may cooperate with antigen heterogeneity to permit the persistence of resistant tumor subpopulations.

Metabolism may also influence the immune component of ADC activity and the efficacy of ADC-immunotherapy combinations. Lactate-rich, hypoxic, and acidic microenvironments can suppress cytotoxic T-cell function and reinforce immunosuppressive networks (Wang et al., 2020; Kepp and Kroemer, 2025). In addition, nutrient competition between tumor cells and immune cells can restrict T-cell metabolic fitness and effector function (Chang et al., 2015). Because ADCs such as EV may induce immunogenic cell death and promote antitumor immune activation, these metabolic barriers may reduce the conversion of payload-mediated tumor-cell death into durable immune responses and may weaken synergy with ICIs. Thus, metabolic processes in BC may affect ADC response through interconnected effects on drug delivery, target engagement, endolysosomal processing, payload sensitivity, bystander killing, and immune activation. At present, however, metabolic regulation of ADC resistance remains biologically plausible but insufficiently validated in BC, and should therefore be interpreted as a BC-relevant hypothesis rather than an established clinical resistance pathway.

## 3 Strategies to overcome ADC resistance

**FIGURE 2:**
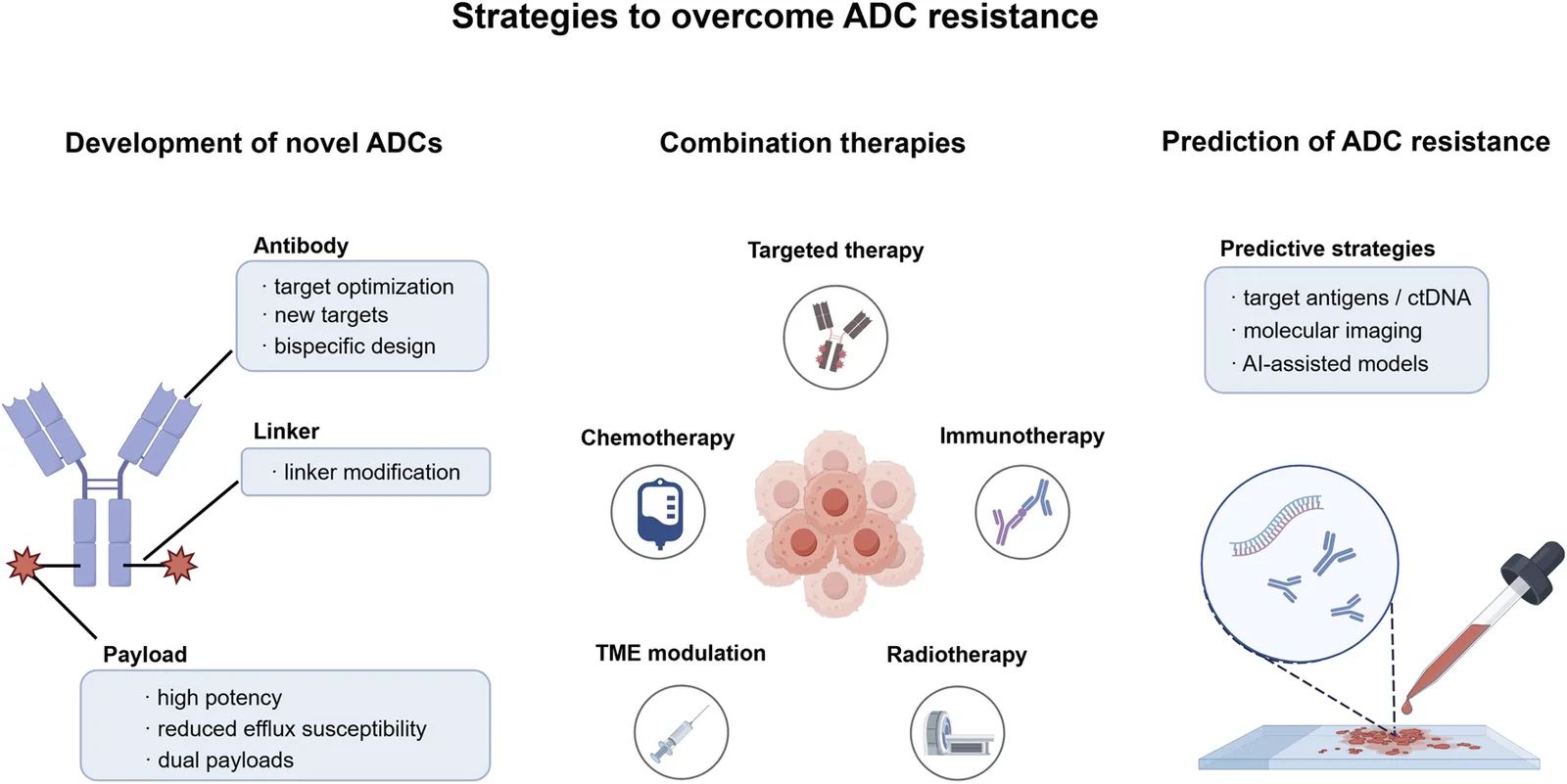
Schematic overview of the principal strategies to overcome resistance to ADCs in BC, including the development of novel ADCs, combination therapies, and predictive approaches for treatment selection and resistance monitoring. Abbreviations: ADC, antibody–drug conjugate; BC, bladder cancer; ctDNA, circulating tumor DNA; AI, artificial intelligence; TME, tumor microenvironment.

To address ADC resistance in BC, therapeutic strategies should extend beyond simply intensifying treatment and instead focus on mechanism-informed optimization (Figure 2). Based on the resistance pathways discussed above, current efforts can be broadly grouped into three complementary directions: the development of next-generation ADCs, rational combination strategies, and predictive approaches to identify patients most likely to benefit from treatment. Together, these strategies may provide the foundation for more effective, durable, and individualized ADC-based therapy in BC.

### 3.1 Development of novel ADCs

To overcome ADC resistance in BC, the development of next-generation ADCs is being pursued across multiple dimensions, including optimization of target selection, modification of payloads and linkers, and innovation in antibody formats (Zhou X., 2025). In the development of novel ADC agents, optimization and expansion of target selection constitute a major strategic focus for overcoming resistance and improving therapeutic efficacy. Current efforts focus on both refining established targets and identifying novel antigens with greater tumor specificity, higher internalization efficiency, or an ability to better address target heterogeneity (Jiang et al., 2024; Chen B. et al., 2025). Nectin-4 is currently the most established target for ADC therapy in BC; however, its heterogeneous expression may contribute to therapeutic resistance (Chu et al., 2021). At the basic research level, current efforts are focused on maximizing the efficacy of ADCs directed against existing targets through a variety of strategies. As mentioned earlier, targeting sialylation enhanced the therapeutic activity of EV in BC, both alone and in combination with immunotherapy. This effect may be attributable to reversal of α2,6-sialylation-mediated Nectin-4 downregulation together with increased EV endocytosis and enhanced EV-induced immunogenic cell death (Wu et al., 2026). In addition, studies have shown that inhibition of autophagy can significantly enhance the antitumor activity of Nectin-4-MMAE (Wang et al., 2024). These findings provide potential strategies for overcoming resistance to Nectin-4-targeted ADCs. Trop-2 is another highly expressed target that has been extensively investigated in BC, and Trop-2-targeted ADCs have already demonstrated clinical activity in UC. This not only supports the therapeutic feasibility of targeting Trop-2 in BC but also provides a rationale for the further optimization of next-generation ADCs directed against this target (Loriot et al., 2024b; Necchi et al., 2026). Recently, Cui and colleagues developed a novel ADC targeting FGFR3, an antigen highly relevant to BC, and preclinical studies have demonstrated promising efficacy (Cui et al., 2026). FGFR3-directed ADCs also have translational relevance because FGFR3 is already an actionable clinical target in UC. Erdafitinib, an oral FGFR inhibitor, is approved for FGFR3-altered locally advanced or metastatic UC after prior systemic therapy, and the phase III THOR trial showed improved survival compared with chemotherapy in this molecularly selected population (Loriot et al., 2023; Guercio et al., 2023). Although erdafitinib is not an ADC, these data support the feasibility of FGFR3-directed therapeutic strategies in UC. At present, multiple novel targets with high and selective expression in BC and favorable internalization properties have entered the field of ADC development. The exploration of these emerging targets is expected to provide new therapeutic options for patients who are resistant to existing treatments (Wei et al., 2025).

In terms of payload strategy, to address resistance caused by upregulation of multidrug resistance proteins, next-generation ADCs tend to employ nonclassical microtubule inhibitors or DNA-damaging agents as payloads. For example, although EV, which targets Nectin-4, uses the conventional microtubule inhibitor MMAE as its payload, subsequent studies on EV and newer ADCs directed against targets such as Trop-2 are exploring payloads including DNA topoisomerase I inhibitors. Owing to their distinct mechanisms of action, these payloads are less susceptible to efflux by ABC transporters and can generate a potent bystander effect while inducing DNA damage, thereby facilitating the elimination of tumor cell populations with antigen heterogeneity (Roth et al., 2026; Tarantino et al., 2022). In addition, site-specific dual-payload ADCs have also been developed. These ADCs are designed to deliver two cytotoxic agents with distinct mechanisms of action to tumor cells through the same antibody molecule, with the aim of synergistically overcoming resistance and enhancing therapeutic efficacy (Wen et al., 2025). Notably, dual-payload ADCs represent an emerging concept that remains at an early exploratory stage, and their therapeutic advantages still require further validation and optimization in preclinical and clinical studies.

In terms of antibody engineering, bispecific ADCs have become a major focus of current development. In BC, this includes the design of bispecific ADCs capable of simultaneously binding two distinct targets, such as Nectin-4 and Trop-2. Such a strategy may offer several advantages. First, it may enable broader coverage of tumor cell populations, thereby reducing resistance caused by the loss of a single target. Second, bivalent binding may enhance the stable association of ADCs with the cell membrane and promote more efficient internalization and lysosomal trafficking, thereby increasing intracellular drug concentrations in tumor cells. In parallel with the development of true bispecific ADCs, dual-ADC combination strategies have also entered early clinical evaluation. The safety and efficacy of the dual-ADC combination strategy (SG plus EV) have already been evaluated in a phase I clinical trial in metastatic UC (NCT04724018) (McGregor et al., 2024). At present, the development of such bispecific ADCs in BC remains largely at the preclinical stage, but they are already regarded as highly promising next-generation technologies. In addition, to improve the penetration of ADCs into solid tumor tissues, structural and functional optimization of specific antibody domains has become an important area of investigation. According to current advances in antibody fragment engineering, the rational optimization of the Fc region in ADC design may enhance the ability of these agents to traverse the vascular wall and penetrate dense tumor stroma (Saunders, 2019).

Finally, artificial intelligence-assisted approaches may further accelerate the development of next-generation ADCs by improving target prioritization, antibody engineering, and linker-payload optimization. Although their application in BC-specific ADC development remains at an early stage, these strategies may support the rational design of ADCs with enhanced efficacy and reduced toxicity (Wang R., 2025). In summary, the development of next-generation ADCs for BC requires a systematic engineering approach. By optimizing target selection, incorporating novel payloads, and exploring improved antibody architectures, such efforts aim to overcome existing resistance mechanisms in a comprehensive manner. Some of these approaches have already entered the preclinical or early clinical evaluation stage, offering new hope for ADC-based therapy in BC.

### 3.2 Combination therapies

Combination therapy is one of the most clinically advanced strategies for improving the efficacy of ADCs in BC, but different combinations vary substantially in the strength of supporting evidence. Therefore, these strategies should be considered according to evidence level rather than discussed as a single category. The most clinically validated example is EV plus pembrolizumab. Mechanistically, ADCs may enhance antitumor immunity by inducing immunogenic cell death, promoting tumor antigen release, and increasing immune recognition, thereby providing a rationale for combination with ICIs (Nicolò et al., 2022; Zippelius et al., 2025). Clinically, the EV-103 study showed encouraging activity of EV plus pembrolizumab in previously untreated advanced UC, leading to accelerated approval in cisplatin-ineligible patients (O’Donnell et al., 2023). Subsequently, the EV-302 trial demonstrated superior survival outcomes with EV plus pembrolizumab compared with platinum-based chemotherapy, establishing this regimen as a practice-changing first-line treatment option for patients with advanced UC (Powles et al., 2025a). This regimen therefore represents the clearest clinical proof of principle that ADC-based combinations can improve outcomes in BC.

Other ADC-based combinations remain clinically promising but are supported by less mature evidence. The combination of ADCs with chemotherapy represents a direct strategy to combine broad cytotoxic pressure with target-directed payload delivery. For example, DV combined with gemcitabine has shown favorable clinical activity in MIBC (Huang H. et al., 2025). The potential rationale is that chemotherapy may increase cellular stress, alter antigen expression, or complement ADC-mediated killing of antigen-positive tumor subpopulations. However, compared with EV plus pembrolizumab, these regimens remain less established in BC and require further prospective validation. Their clinical use will also need careful consideration of overlapping toxicities, including myelosuppression, hepatic dysfunction, and peripheral neuropathy (Wei et al., 2024).

In contrast, combinations designed to overcome specific resistance mechanisms, such as impaired intracellular trafficking, lysosomal dysfunction, metabolic adaptation, or TME-mediated immune suppression, remain largely preclinical or hypothesis-generating. Agents that restore lysosomal processing, improve ADC internalization, modulate target antigen expression, reduce stromal barriers, or reverse immunosuppressive and metabolically hostile microenvironments may theoretically enhance ADC efficacy (Wei et al., 2024; Zhou K. et al., 2025). However, these strategies should be distinguished from clinically validated regimens because direct evidence in ADC-treated BC cohorts remains limited. Future combination strategies should therefore be guided by mechanism-based biomarkers, rational sequencing, and prospective clinical trials rather than empirical treatment intensification.

### 3.3 Prediction of ADC response and resistance

The pre-identification of patients who are unlikely to respond to a specific ADC therapy through predictive biomarkers is a key strategy for achieving precision medicine, avoiding ineffective treatment, and optimizing the allocation of medical resources (McNamee et al., 2026). The expression and characteristics of target antigens constitute the cornerstone of efficacy prediction. For example, studies have shown that Nectin-4 amplification is a simple and valuable predictive biomarker for EV treatment in patients with metastatic UC, and high Nectin-4 protein expression on BC cells, as determined by immunohistochemistry, has been confirmed to be associated with better therapeutic responses (Klümper and Eckstein, 2024; Klümper et al., 2024). However, the efficacy of ADCs is often limited by intratumoral heterogeneity in molecular expression. Membranous Nectin-4 expression frequently decreases during metastatic dissemination of UC, suggesting that tissue-based expression levels alone may be insufficient for response prediction. It has therefore been proposed that pretreatment biopsy of metastatic lesions may improve the prediction of response to EV (Klümper et al., 2025). In the field of nuclear medicine, Ren et al. proposed an iodine-labeled anti-Nectin-4 ADC positron emission tomography (PET) probe that may help address the issues described above. This probe conjugates a Nectin-4-targeting antibody with a positron-emitting radionuclide, such as iodine-124, with the aim of enabling theranostic integration (Ren et al., 2024). In addition, Duan et al. also proposed a Nectin-4-based radioligand, 68Ga-N188, as a PET-assisted diagnostic tool and conducted related translational studies in patients (Duan et al., 2023). These PET imaging approaches may enable noninvasive and quantitative whole-body mapping of Nectin-4 expression, thereby providing a prospective means of assessing the suitability of ADC therapy. Furthermore, serial imaging during treatment may allow visualization of ADC accumulation and retention at tumor sites, which could support early evaluation of therapeutic response and help elucidate resistance mechanisms such as antigen downregulation, ultimately guiding individualized treatment optimization. Notably, circulating tumor DNA (ctDNA) is an emerging biomarker with the potential to support personalized sequencing strategies, and its predictive value in ADC resistance warrants urgent investigation. Finally, integration of multiple biomarkers through artificial intelligence and spatial analysis is expected to substantially improve predictive performance beyond that of any single biomarker (Coca Membribes et al., 2026).

## 4 Conclusion and perspectives

ADCs have expanded the therapeutic landscape of BC, but primary and acquired resistance remain major barriers to durable clinical benefit. Current evidence indicates that ADC resistance in BC is shaped by target antigen heterogeneity, impaired intracellular processing, altered payload sensitivity, tumor heterogeneity, and microenvironmental barriers. Metabolic processes should be viewed as cross-cutting modulators rather than as an isolated resistance category, because they may influence ADC delivery, intracellular processing, payload sensitivity, bystander killing, and immune synergy.

A key message of this review is that the strength of evidence varies substantially across resistance mechanisms. BC-specific evidence is strongest for target-related resistance, particularly in the context of Nectin-4 and EV, whereas several mechanisms, including ABCB1/P-glycoprotein-mediated payload efflux, lysosomal dysfunction, impaired trafficking, and metabolic regulation, remain incompletely validated in BC-specific models and clinical cohorts. Distinguishing established mechanisms from biologically plausible hypotheses is therefore essential for avoiding overinterpretation and for defining priorities for future research.

Clinically, the most advanced strategy for improving ADC outcomes in BC is rational combination therapy, especially ADC-immunotherapy combinations such as EV plus pembrolizumab. Additional priorities include next-generation ADC design, biomarker-guided patient selection, rational sequencing, and prospective analyses of paired pre- and post-treatment samples. Together, these approaches may help translate mechanistic insights into more durable and individualized ADC-based therapy for BC.

## Glossary

ABC: ATP-binding cassette
ABCB1: ATP-binding cassette subfamily B member 1
ADC: antibody–drug conjugate
AI: artificial intelligence
APLP2: amyloid precursor-like protein 2
BC: bladder cancer
CAV1: caveolin-1
CDE: caveolae-mediated endocytosis
CME: clathrin-mediated endocytosis
ctDNA: circulating tumor DNA
DV: disitamab vedotin
EV: enfortumab vedotin
FGFR3: fibroblast growth factor receptor 3
HER2: human epidermal growth factor receptor 2
HMGB1: high-mobility group box 1
ICI: immune checkpoint inhibitor
la/mUC: locally advanced or metastatic urothelial carcinoma
MDSC: myeloid-derived suppressor cell
MIBC: muscle-invasive bladder cancer
MMAE: monomethyl auristatin E
Nectin-4: nectin cell adhesion molecule 4
NMIBC: non-muscle-invasive bladder cancer
ORR: objective response rate
OS: overall survival
PD-1: programmed cell death protein 1
PD-L1: programmed death-ligand 1
PET: positron emission tomography
PFS: progression-free survival
P-gp: P-glycoprotein
SG: sacituzumab govitecan
T-DM1: trastuzumab emtansine
TAM: tumor-associated macrophage
TMB: tumor mutational burden
TME: tumor microenvironment
TOP2A: DNA topoisomerase II alpha
Treg: regulatory T cell
Trop-2: trophoblast cell surface antigen 2
TURBT: transurethral resection of bladder tumor
UC: urothelial carcinoma
V-ATPase: vacuolar H + -ATPase
